# Outward looking foreign direct investment and its determinants in highly indebted Eastern African countries

**DOI:** 10.1371/journal.pone.0297142

**Published:** 2024-02-01

**Authors:** Habtamu Getachew Tegegne

**Affiliations:** Department of Accounting and Finance, Woldia University, Woldia, Ethiopia; Palestine Technical University - Kadoorie, STATE OF PALESTINE

## Abstract

This study examines outward-looking foreign direct investment (FDI) and the determinants of four highly indebted low-income countries in East Africa. To achieve the stated objective, the study utilizes the pooled mean group (PMG) approach for panel data encompassing the period from 1990 to 2022. Additionally, bound testing and the autoregressive distributed lag (ARDL) model are applied to analyze time series data from individual countries within the sample. The panel PMG/ARDL estimation suggests that both market size and exchange rate have a significant positive impact on FDI inflows, both in the short run and the long run. Specifically, the time series analysis using ARDL estimation reveals that market size has a positive and significant impact on FDI inflows for both Rwanda and Tanzania, both in the long run and the short run. Furthermore, the association between the labor force and FDI inflows is positive only in the long run for Rwanda, while for Tanzania, it shows a positive association in both the short run and the long run. In terms of the availability of natural resources, the analysis indicates a positive impact in the short run but a negative association with FDI inflows in the long run, with the exception of a positive association with Rwanda in the long run. Additionally, external debt has a positive and significant impact on FDI inflows for Kenya, both in the short run and the long run. Based on the findings, the study recommends that policymakers should focus on policies and strategies that promote market expansion and create a larger consumer base. This can be achieved through initiatives such as market development programs, trade agreements, and regional cooperation.

## Introduction

After the wake of World War II, the subject of foreign direct investment (FDI) is currently a hot topic both nationally and internationally [1]. FDI flow continues to be an imperative source of external finance for emerging economies [2]. In the twenty-first century, foreign direct investment (FDI) is growing at an astounding rate [3]. According to [4], global competition in the hope of attracting FDI provoked the host governments to tear out their entrance requirements, taxes, environmental approvals, and working condition requirements. Since FDI is a powerful tool for combating poverty [5] and boosts economic growth through the spread of technology, development of human capital, promote of export, creation of job opportunities, and productivity growth [6–11]. Indeed, FDI is a vital ingredient in achieving sustained growth of any nation and the dominating factor that helps to propel the economic growth [12]. As such, World FDI flows were $1.58 trillion in 2021, up 64% from the amount of less than $1 trillion in the first year of the COVID-19 epidemic. Due to growing merger and acquisition markets, rapid expansion in international project finance, lenient financing conditions, and big infrastructure stimulus packages, FDI flows appear to be gaining significant speed [2]. FDI flows to developing economies in 2021 increased by 30% $837 billion, the highest level ever recorded.

FDI can significantly contribute to Africa’s development struggles, including: boosting domestic savings, creating jobs and integrating into the global economy, transferring cutting-edge technologies, FDI can act as a catalyst for economic diversification, assisting African nations to escape an unhealthy dependency on natural resources [13]. Further [14], stated that FDI initiatives in Africa had a substantial effect on SSA economic growth. From $39 billion in 2020, FDI flows to Africa reached $83 billion, a record high, and accounted for 5.2% of global FDI. Following the decline in FDI brought on by the pandemic in 2020, most recipients had a modest increase. Despite the fact that African countries have worked hard to entice FDI, the volume of FDI that flows into Africa, mainly SSA, is very low compared to other part of the world [15, 16]. The results were not fruitful [17].

With $8.2 billion, FDI in East Africa increased by 35%. To Ethiopia, $4.3 billion was transferred. Ethiopia serves as the focal point for China’s Belt and Road Initiative, and Chinese investments. Uganda which has the next FDI distinction, rose by 31% to $1.1 billion and Tanzania rose by 35 per cent to $922 million [2]. Nevertheless, in 2022, when the globe was still feeling the effects of the pandemic, the conflict in Ukraine broke out, drastically altering the global environment for international trade and cross-border investment. The war is having an impact far beyond its local area, resulting in a triple food, fuel, and financial crisis, with inflation driven by rising energy and basic commodity costs and spiraling debt getting worse. Furthermore, macroeconomic variables like rising interest rates, are projected to have a significant impact on FDI flows to developing nations in 2022. The effects of increasing energy prices on local consumption, high food prices, which can cause political instability, and tighter financial conditions, are the key factors contributing to a potential decline in FDI, Global FDI in 2022 may experience significant downward pressure due to investor uncertainty and risk aversion [18].

Furthermore, for over 20 years, a number of the poorest countries in the world have been plagued by doubt over hang. The debt overhang can act as a tax on investment results, discouraging savings and investment. Heavily indebted countries tend to really heavily on trade taxes which can be easily collected at the port or exit. High tax revenue therefore translated in to high taxes which adversely affect FDI investors. Finally, the cumulative effects pose a risk of default and discourage investment [19].

According to [20], FDI inflow to the Africa continent are unevenly spread and mostly concentrated in oil exporting nations. As such, the size of the economy and oil petroleum exporting are a significant contributions to FDI inflow. However, Study by [21] suggested that natural resources (oil and gas) were negatively associated with FDI in 16 MENA regions. The result of the study is contrary to resource seeking FDI. While market size and natural resource endowment have prominent significant contribution to FDI in the region, the result supports the market seeking motives of FDI. Whereas [22] stressed that natural resource endowment is not enough to attract FDI, rather, the implementation of NEPAD program will spur FDI inflow over the African continent. Yet [23], proposed, Africa is dominated by neither export seeking nor market seeking motives of foreign direct investors. On the other hand [24], in his study stressed that China FDI attracted to invest in Africa due to the large market size and availability of natural resources.

### Outward looking FDI in flow in highly indebted East African countries

**Fig 1:**
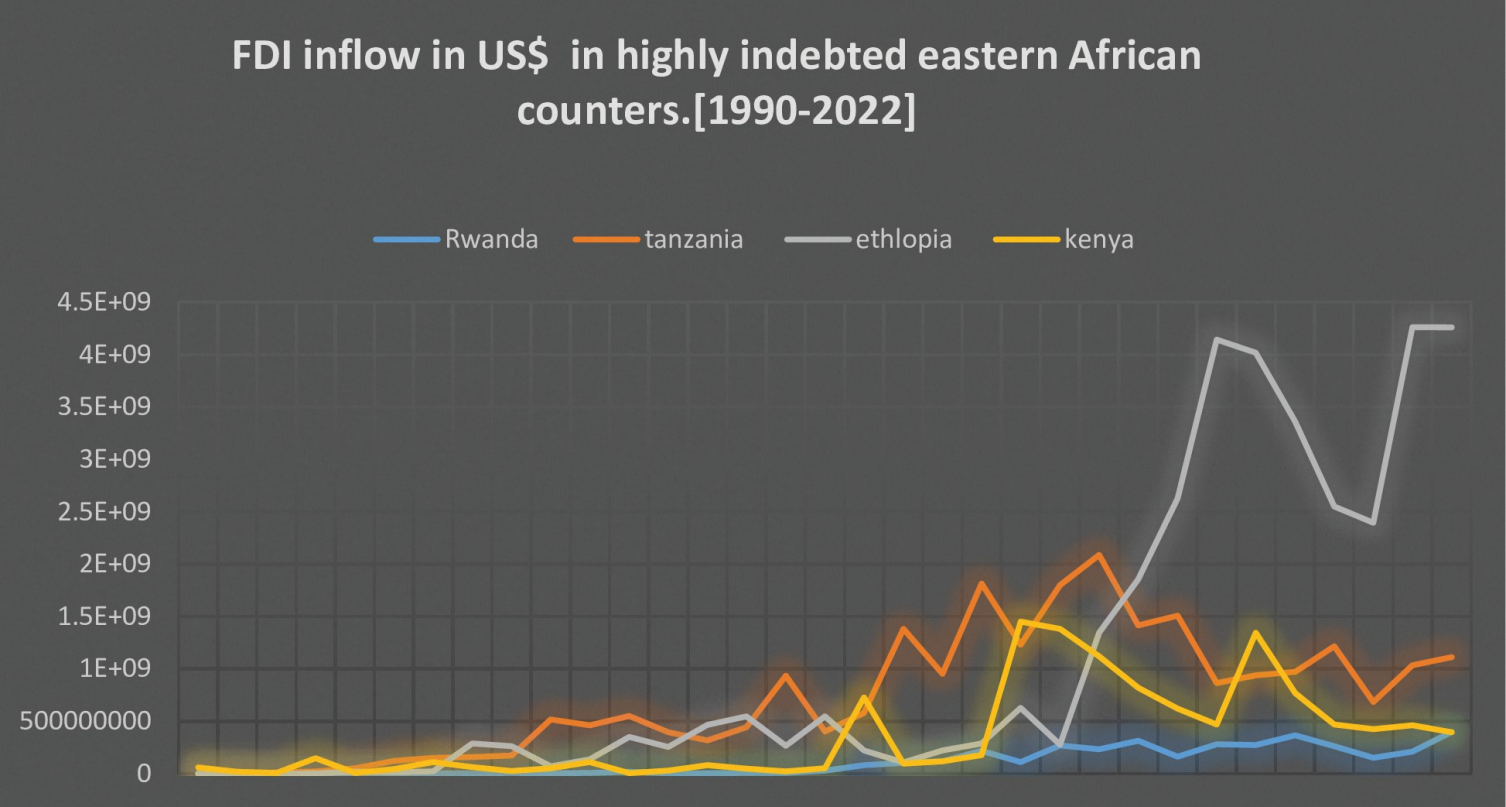
FDI in flow in US$ (1990–2022).

In 2021, Africa experienced a significant surge in foreign direct investment (FDI), reaching a record-breaking level of $83 billion, up from $39 billion in 2020. This accounted for 5.2% of the global FDI. FDI flows in Southern Africa, East Africa, and West Africa saw notable increases, while Central Africa remained stable and North Africa experienced a decline. East Africa, in particular, saw remarkable growth with FDI soaring by 35% to reach $8.2 billion. Ethiopia emerged as a frontrunner among the least developed countries in East Africa, attracting FDI worth $4.3 billion, largely due to its involvement in China’s Belt and Road Initiative. The United Republic of Tanzania also experienced a substantial 35% increase in FDI, reaching $922 million, accompanied by a surge in new Greenfield project announcements. Notable projects included a nickel project by Kabanga Nickel (United Kingdom) worth $318 million and an investment in the food and beverage industry by Associated British Foods (United Kingdom) worth $238 million [18] (Fig 1).

Previous studies have primarily examined the determinants of foreign direct investment (FDI) in African nations as a whole/individually, inadvertently overlooking the motives and determinants of FDI in highly indebted Eastern African countries when employing both PMG approach and the ARDL model. In light of this gap, this study aims to conduct a more focused investigation into the factors that influence FDI in Eastern African nations grappling with substantial debt burdens. By addressing these contextual and methodological gaps, the study has the potential to deepen our comprehension of the determinants of FDI in highly indebted Eastern African countries, thereby offering valuable insights for policymakers and practitioners operating within this specific context. Therefore, it is found sound to examine the key motives and determinants depending on the level of external debt for highly indebted LDCs, particularly Eastern Africa.

### Contributions and objectives of the study

Overall, the study provides a substantial contribution to the comprehension of the determinants of outward-looking foreign direct investment (FDI) in highly indebted developed East African countries. The findings of the study hold valuable insights for policymakers, regulators, and other stakeholders, enabling them to make informed decisions and take appropriate actions to attract FDI in the East Africa region, with a particular focus on the least developed countries (Ethiopia, Rwanda, Tanzania, and Kenya).

The main objective of this study was to examine the outward looking foreign direct investment (FDI) determinants of highly indebted African counters.

### Hypothesis of the study

H01 = there is no relationship between market size, inflation, labour cost, availability of natural resources, exchange rate, and external debt with foreign direct investment.

### Literature reviews

The fundamental problems associated with FDI in Sub-Saharan Africa have become clear over the last decade, as the investment is heavily influenced by macroeconomic shocks that have severely impacted FDI inflows due to changes in macroeconomic factor [25]. The following subsections discuss both theoretical and most recent empirical research on motives and key determinates of FDI.

### Theoretical and empirical reviews

Various researchers and schools of thought have put forth numerous ideas to explain Foreign Direct Investment (FDI). Prominent theories in this field have emerged since the post-World War II era, with contributions from various researchers such as [26–31], and others. However, there is no consensus on a comprehensive hypothesis that effectively explains FDI. The Heskscher-Ohlin model, a key aspect of neoclassical trade theory, suggests that trade opportunities and capital movements between nations depend on the relative availability of production factors. Consequently, multinational corporations strategically invest in countries to take advantage of higher investment returns or lower production costs.

Market imperfection theory suggests that multinational corporations may establish their businesses or manufacturing operations in certain nations due to market imperfections, with the aim of utilizing economies of scale, ownership advantages, and government incentives [29]. [32], Product Life-Cycle Theory explains how a new multinational corporation introduces and subsequently engages in FDI for a new product. According to the theory, the life cycle stages of a product, from introduction to decline, influence the decision of an MNC to either export the product or establish production facilities in foreign markets. The MNC’s objective is to minimize manufacturing costs while satisfying the growing demand for its products in both domestic and foreign markets at a competitive price.

According to Vernon’s Product Life-Cycle Theory, a new product is initially manufactured in its home country to cater to the domestic market. As the domestic market becomes saturated and faces intense competition, the product is then exported to other countries. In later stages, producers actively seek lower-cost foreign locations to continue production. This theory elucidates how multinational corporations pursue market expansion and cost reduction objectives through FDI. It also sheds light on the behavior of these corporations and how they take advantage of countries at different stages of development. Additionally, Vernon suggests that firms use foreign direct investment as a defensive strategy to protect their existing market position [33].

[27, 34, 35] the eclectic paradigm is likely the most thorough theoretical stance for justifying multinational corporations’ decisions to engage in FDI. Firms invest in FDI for three reasons: ownership advantage, locational advantage, and internalization (OLI). The ownership feature can provide a corporation with control over resources, technology, or financial capital and allow firms to compete with other firms regardless of whether they are foreign. Location advantage is defined by several key factors, including access to large domestic markets, abundant natural resources, a skilled and educated labor force, low labor costs, robust institutions, political stability, and favorable tax rates [36]. Found that natural resource endowment and market size positively impact FDI in landlocked African nations. Furthermore [37], suggested that a large domestic market size is a prominent driver of FDI in Africa, particularly in SSA and MENA countries.

Contrary to previous findings, a study by [38] on 22 SSA nations indicates that market size has a negative and limited long-term impact on FDI. Additionally [39], suggests that market size does not have a clear relationship with FDI in Ethiopia. Investors aim to maximize their return on investment, and the market size of the host nation is influenced by economic standards, exports, and imports. Countries experiencing growth in these areas have the potential to attract significant foreign investment [36].

Bundled FDI offers superior commercial gains through internationalization advantages, leveraging ownership-specific and location-specific advantages. The Eclectic paradigm recognizes that OLI parameters vary among firms, shaped by the context and economic, political, and social aspects of the host country [40]. Market-seeking (horizontal) FDI aims to replicate production facilities in the host country to better serve the local market and bypass tariffs, considering factors like market size and expansion potential. However, barriers to accessing local markets can discourage firms from pursuing this type of FDI. Chinese investors in Africa are often driven by resource seeking rather than efficiency and market seeking [41]. Developed western foreign investors typically target economies with large markets, as market size has the power to attract FDI [42]. Studies by [10, 43–48] support the idea that a large domestic market size is a prominent driver of FDI in Africa, particularly in SSA and MENA countries. In contrast [11], found that market size plays an insignificant role in attracting FDI, and investors in Rwanda are not primarily seeking market-oriented FDI.

Resource-seeking (vertical) or export-oriented FDI involves multinational corporations investing in foreign countries to gain access to essential resources such as raw materials, labor, and natural resources. This type of FDI often entails establishing interconnected production processes in the host country. The decision to invest in resource sectors is typically driven by factors like the availability of low-cost labor and abundant natural resources. In Sub-Saharan African countries, the attraction of FDI is still influenced by the presence of natural resource endowment, as highlighted by [49]. The positive impact of natural resources on FDI stems from the increased value of re-investible reserves, as mining and exploration companies operate in the region. This, in turn, aligns with the theoretical expectation that competition for ownership and control motivates parent firms to invest in foreign subsidiaries. Another study [50], suggests that the availability of natural resources can have a positive impact on attracting FDI. For example, Morocco, Egypt, and Tunisia witnessed significant FDI inflows in 1999 due to their resource availability, and Angola received the largest volume of FDI in 1998, primarily driven by its oil resources. However, it is important to note that simply having abundant natural resources may not be sufficient to attract FDI, as resource endowment can be affected by measurement-related issues, as pointed out by [49]. Overall, the availability of natural resources plays a substantial role in influencing foreign direct investment (FDI) decisions in host countries, particularly in the case of resource-seeking FDI.

When a country has abundant natural resources but lacks the necessary capital or technical expertise to exploit them and sell them globally, foreign direct investment (FDI) becomes necessary. Foreign companies engage in vertical FDI within the host country to acquire the raw materials or inputs needed for their production processes in their home countries. In such cases, the decision to invest is often driven more by the presence of inaccessible natural resources rather than the host country’s profitability or market size. A study conducted by [51] found that market size has a significant positive impact on attracting FDI in BRICS nations, while the availability of natural resources has a negative impact. This suggests that most investors in BRICS countries are motivated by seeking market opportunities and acquiring essential resources. Additionally, another study suggests that, with all else being equal, countries with abundant natural resources tend to attract more FDI [52].

Efficiency-seeking FDI is the third type, which aims to achieve economies of scale and scope by corporations. They seek to benefit from the shared governance of geographically dispersed activities. The primary motivation for this type of FDI is often the potential for cost efficiency and risk diversification. An empirical study conducted on the motives of FDI in Sub-Saharan Africa [53] revealed that strategic and efficiency-seeking factors influenced inward FDI in SSA between 1996 and 2010. Market size also had an influence on FDI, although its impact was less robust across different specifications. Surprisingly, the study found that FDI in SSA was not primarily driven by resource-seeking motives. In West and Central Africa, FDI was driven by efficiency and market-seeking motives, while South and East Africa were predominantly characterized by efficiency-seeking motives. Another study conducted on FDI in Oman from 1980 to 2013 found that market size and natural resources had a long-term positive relationship with FDI [50]. The author concluded that the flow of FDI in Oman was characterized by both market-seeking and resource-seeking motives.

The location-based approach suggests that the social, economic, and political conditions of a host country determine the level of foreign direct investment (FDI). Countries with natural resources, competent labor, business-friendly policies, physical infrastructure, and a large market tend to attract FDI [54]. Microeconomic factors, such as a firm’s motives for location, also play a role, but macroeconomic factors are considered in the overall decision-making process. Studies have shown that GDP growth rate and resource availability positively influence FDI in African nations. Market size, availability of natural resources, GDP growth, and labor force growth have been found to have a statistically positive impact on FDI inflow in a study covering 88 countries [46]. Different types of resource-seeking FDI are influenced by physical resources and a plentiful supply of cheap labor [40]. Market size and exchange rate have been identified as significant factors in attracting FDI in Asian nations, while labor productivity is a more important consideration for foreign investors than cheap labor [44]. Factors such as market size and the quality of the labor force impact FDI attraction in specific cases like Mauritius [55]. Higher GDP and growth rates have been found to attract FDI in both developed and developing nations [56].

The institutional FDI fitness theory, developed by [30], states that four pillars are necessary for attracting and benefiting from foreign direct investment (FDI): effective government, a free market, investment in education, and socio-cultural norms that promote investment. Unlike the location-based approach, which focuses on physical resources, this theory emphasizes the importance of institutional factors such as government policies, educational programs, and cultural norms as determinants of FDI. Countries that prioritize businesses and education tend to attract higher levels of FDI. The concept of FDI fitness, introduced by [30], refers to a country’s ability to attract, absorb, and retain FDI. The theory suggests that government, market, educational, and socio-cultural fitness are all essential components for FDI attractiveness. Sociocultural variables are considered foundational in this framework. The theory aims to explain the uneven distribution of FDI flows among countries.

The capital market theory, also known as the "currency area theory," suggests that foreign investment, including foreign direct investment (FDI), is influenced by imperfections in the capital market, particularly differences in currency strength between the home and host countries [28]. Host nations with weaker currencies are more likely to attract FDI compared to those with stronger currencies. However, this theory has received criticism for its limited ability to explain investment patterns in countries with strong currencies [57]. Studies conducted in Africa have shown that exchange rates can have a negative impact on FDI inflow, while economic growth has a positive effect. Depreciation in exchange rates has varied impacts on FDI in different African countries. Some studies indicate a positive association between exchange rates and FDI, while others suggest that currency devaluation can improve the purchasing power of foreign investors [58]. The relationship between exchange rates and FDI varies across different countries and time periods. In some cases, currency appreciation in the host country can discourage FDI, as it makes the country’s assets less attractive to foreign investors. Fluctuations in exchange rates also introduce uncertainty in export prices.

Investors are generally reluctant to invest in an environment with high inflation pressure due to its association with economic instability and weak monetary management. Inflation can negatively impact foreign direct investment (FDI) by eroding the profitability of multinational corporations and creating uncertainty regarding investment returns. A study supported by [49] found that inflation can have a detrimental effect on FDI as it erodes the investment’s potential returns. However, another study focusing on East Africa [59] found that economic growth, exchange rates, and inflation had a positive but statistically insignificant impact on FDI flows in the region. This suggests that macroeconomic factors have limited influence on investment decisions in Eastern Africa.

According to [60], Kenya can attract more foreign direct investment (FDI) by reducing external debt and achieving higher GDP, which increases investor confidence. In the ECOWAS region, a study by [43] covering the period 1985–2015 found that exchange rate, economic growth, and market size are key determinants of FDI. Another study focusing on the MENA region by [61], analyzing the years 1970–2010 using panel co-integration tests, concluded that higher economic growth rates lead to increased FDI inflows. [10], conducted a study on fast-growing economies in BRICS and MINT countries, and found that market size is a significant determinant of FDI. Communication variables were identified as dominant determinants of FDI in developing countries by [62]. However, the studies did not provide conclusive evidence on the effects of external debt and economic growth on FDI. Additionally, a study by [52] using the ARDL approach on institutional political determinants of FDI in Ethiopia from 1970–2013 found that market size and depreciation in the nominal exchange rate positively influenced FDI inflows.

### The econometric model and data

In order to examine the determinants of FDI in the least developed east African counters, the following econometrics models were developed following [3, 17, 32, 34, 35, 38, 52].


$${LogFDI}_{jt}=\alpha_{j}+\beta_{1j}{LogMS}_{jt}+\beta_{2j}{LogINF}_{jt}+\beta_{3j}{LogLF}_{jt}+\beta_{4j}{LogER}_{jt}+\beta_{5j}{LogNR}_{jt}+\beta_{7j}{LogED}_{jt}+\varepsilon_{jt},$$ Eq (1)

Where *LogFDI*_*jt*_ is the log forging direct investment in flow country j in US$. *LogMS*_*jt*_, log of market size measured by Gross domestic product in current US$. Market size has a significant positive association with FDI [48, 63, 64]. Since a large market tends to attract firms that seek to expand into other markets in order to increase their sales or market share. Additionally, firms may be interested in entering markets that offer potential for growth. In most studies, GDP growth per capita is preferred as an indicator of market growth. Furthermore, market size is identified as a critical determinant of FDI in East Africa [65, 66].

*LogINF*_*jt*_, annual inflation rate measured by annual consumer price index. Macroeconomic stability is a significant factor that influences foreign direct investment. According to [67] inflation does not have an impact on FDI flow. On the other hand, macroeconomic instability has a positive influence on FDI [68].

*LogLF*_*jt*_, labour cost. Cheap labour would reduce the cost of production and limit FDI in flow proxed by wage rate [47]. Africa holds immense potential, primarily due to its abundant labor resources and vast expanse, enabling it to play a significantly more substantial role in the global economy. Nonetheless, as highlighted by [69], African nations must bolster their performance across multiple domains to attract foreign direct investment to the region.

*LogER*_*jt*_, log exchange rate of currency of the host country to the U.S$ devaluation of currency would induce exchange rate risk and improve the purchasing power of the investor in foreign currency terms enhanced. To mitigate the risk of business failure caused by foreign exchange volatility for existing foreign investments in Sub-Saharan African (SSA) countries, it is important to establish exchange rate stability among these nations [70, 71]. *LogNR*_*jt*_, In many countries rich in natural resources, the common practice is to export these resources in their raw form to developed countries, where the cost of labor tends to be higher compared to developing countries. Consequently, investors face high production costs in regard to processing these raw materials. This situation acts as a deterrent to foreign direct investment (FDI) inflow [65]. Natural resources provide an absolute advantage to a country because their abundance is often associated with factors of production. However, beyond a certain threshold, an increased level of natural resources can have a negative and significant impact on foreign direct investment [72]. Conversely [67, 70], argues that the mere presence of natural resources in Africa does not contribute to the attraction of FDI. *LogED*_*jt*_, Log of external (foreign) debt measured by US$ expects a positive association between external debt and financial development. and *ε*_*jt*_, the white noise error term.

Panel data afford more degrees of freedom through extra decisive and effectual estimates comparably for all sample developed counters. According to [73]. Panel long and dynamic short run relationship among variables estimated using PMG (pooled mean group) approach. It analysis takes the co-integration form of the simple ARDL model and allows the error variance and intercept to differ across cross-section entities but limits the long-run coefficients (β) to be the same using an error correction framework.

In order to examine market size, inflation, labour costs, exchange rate, and external debt effects on foreign direct investment, the given (Eq 2)

Z=[LogMS,LogINF,LogLC,LogER,LogNRandLoED] The PMG/ ARDL model is stated as

$$\Delta Log{FDI}_{i,t}=\alpha_{i}+\phi_{i}Log{FDI}_{i,t-1}+\beta_{i}LogZ_{i,t-1}+\sum_{j=1}^{\rho -1}\lambda_{ij}\Delta Log{FDI}_{i,t-j}+\sum_{j=0}^{\sigma -1}\delta_{ij}\Delta LogZ_{i,t-j}+\varepsilon_{i,t,}$$ Eq (2)

Where *α*_*i*_ is country-specific intercepts (*α*_*i*_); *ϕ*_*i*_ is the adjustment coefficient; and *θ* = −(*β*_*i*_/*ϕ*_*i*_) is long-run coefficient which is defined to be the same across the group.

Thank you. The study utilizes the ARDL approach with a VEC model as the foundation for analyzing the dynamics of foreign direct investment (FDI) in various African contexts, particularly in highly indebted Eastern African nations. The ARDL model, a time series econometric approach, captures both short-term and long-term relationships between variables. In this study, the model incorporates specific characteristics and considerations that are relevant to this region, aiming to provide a more accurate and tailored analysis of FDI dynamics in East Africa. Some key aspects of the unique version of the ARDL model for FDI in African countries include the recognition of resource endowments and cheap labor. Africa is renowned for its rich natural resources and low-cost labor force, which can significantly influence FDI patterns. Additionally, the ARDL model for FDI in Africa takes into account factors such as market size, external debt, and external factors like inflation and exchange rates as important determinants.

Additionally, to account for countries’ individual differences, the foreign direct investment equilibrium is estimated for individual cases using time-series data. The study used ARDL approach over VEC estimation for time series analysis. Since it allows different lag orders for both independent and dependent variables in the model, it excludes insignificant lags for all individual variables and it solves the uncertainty whether the variables are stationary at the level or first deference to check long-term association. Besides, the bound test is used to test the long run relationships among variables regardless of whether the variables are stationary at level I (0) or integrated order 1, I (1), but not I(2). Furthermore, the ARDL estimation assumes that all explanatory variables are exogenous, which might cause a bias toward some endogenous explanatory variables.

The bound test was performed to examine the running impact of market size, inflation, external debt, exchange rate, labour force, and availability of natural resources on foreign direct investment. To test the bound test for the existence of long-run relationship among variables in foreign direct investment stated (Eq 3).


$$\Delta Log{FDI}_{i,t}=\mu_{j}+\sum_{i=1}^{\rho }\delta_{i}Log{FDI}_{jt-i}+\sum_{i=0}^{q}\gamma_{i}LogZ_{jtt-i}+\theta_{1j}Log{FDI}_{jt-1}++\theta_{2j}Log{MS}_{jt-1}+\theta_{3j}Log{INF}_{jt-1}+\theta_{4j}Log{ER}_{jt-1}+\theta_{5j}Log{NR}_{jt-1}+\theta_{6j}Log{LF}_{jt-1}+\theta_{1j}Log{ED}_{jt-1}+\varepsilon_{i,t,}$$ Eq (3)

Where, Z=[LogMS,LogINF,LogLF,LogER,LogNR,LogED]

*ρ* = 1,….,*k lags*, *μj* Unrestricted intercept for country j and *ε*_*i*,*t*_, *i* white noise disturbance term for country j

The long-run coefficient values are computed as $\beta_{1}=-(\frac{\theta_{2}}{\theta_{1}}),\beta_{2}=-(\frac{\theta_{3}}{\theta_{1}}),\beta_{3}=-(\frac{\theta_{4}}{\theta_{1}}),\beta_{4}=-(\frac{\theta_{5}}{\theta_{1}})\;and\;\beta_{6}=-(\theta_{7}/\theta_{1})$ for market size, inflation, labour force, exchange rate, natural resource and external debt.

### The data

To examine the determinants of FDI in highly indebted East African countries, annual time-serious data from the years 1990 to 2022 were used, the study period and data chosen purposively based on data availability. The sample nations are selected based on the degree of indebtedness, which is measured as the ratio of external debt to total domestic GDP in current US dollars. The required data were collected and organized from the databases of multilateral institutions including IMF (World Economic Outlook) database and World Bank development indicators.

Macroeconomic and financial time serious studies are conducted based on the assumption that the underline time seriousness is stationary. Stationery indicates the time serious (trend) has a constant mean and bounded (finite) variance, σ2. Stationary test is critical since non stationary data provide superior regression results. As such, the study conducted by Levin, Lin & Chu (LLC), Im, Pesaran and Shin (IPS), Fisher -ADF stationary test for panel data and augmented Dickey-Fuller test for time series data concocted as presented in Table 1, the unit root result shows that some variables are unit root at level I (0) and some variables are stationary at first deference I (1) but neither of the variable is integrated order I (2).

**Table 1:** Unit root tests using LLC, IPS, and ADF–Fisher for panel and ADF for time-series data.

Variables								
Country	Unit root	LogFDI	LogGDP	LogLF	LogED	LogNR	LogINF	LogER
**Panel**								
LLC	level	-16.5383***	0.7358	-1.3334*	2.5903	-1.0554	-2.0654*	-3.9338***
	1st deff.	-12.3485***	-4.0129***	-6.6895***	-5.2967***	-7.9769***	-8.8982***	-4.6298***
IPS	level	0.3105	4.4722	7.7254	2.445	-2.8784***	-1.2272*	4.3809
	1st deff.	-7.4356***	-4.7906***	-5.962***	-4.6702***	-8.3448***	-6.5822***	-3.4116***
Fisher	level	0.7099***	1.0238	0.9957	0.1553***	0.3446***	0.2316***	0.9394
	1st deff.	0.0237***	0.2726***	0.1518***	0.1518***	-0.4686***	-0.5968***	0.2268***
Ethiopia	level	-4.874***	1.497	-1.168	-5.423***	-3.467**	-4.293***	-0.777
	1st deff.	-4.638***	-3.059***	-5.389***	-9.763***	-7.589***	-12.446***	-3.563***
Kenya	level	-3.147**	1.062	-1.793	2.215	-3.07**	-4.761***	-3.312**
	1st deff.	-10.328***	-4.408***	-6.407***	-4.468***	-7.399***	-8.878***	-5.129***
Rwanda	level	-2.399*	-0.086	-0.469	-3.717***	-1.827	-5.42***	-3.369**
	1st deff.	-9.034***	-6.737***	-4.441***	-8.032***	-4.938***	-8.099***	-5.696***
Tanzania	level	-4.797	-0.804	0.154	-4.346	-2.479	-1.83	-5.012
	1st deff.	-5.495***	-4.457***	-7.85***	-9345***	-6.384***	-5.333***	-2.621***

**Note:** SIC was used for Max-lag selection.

*, **, ***Are stationary (unit root rejected) at **10, 5, and 1**% significance levels, respectively

**Source:** Author’s estimation using **STATA 15**

(ADF) for time-series data while Levin–Lin–Chu (LLC) and ADF–Fisher χ2 test for panel data. The unit root results suggested that all data series have unit root, albeit the external debt variable look stationary at the level for some countries, but neither of the variables is I(2). As [74] stated, unit root tests often suffer from poor size and power properties, and thus, it is not compulsory for all variables even in Johansen co-integration estimation to have the same order of integration unless the variables are integrated order 2, I(2).

### Ethical approval and consent for participant

Ethical clearance is not required for this study as it involves secondary data analysis obtained from the World Bank Indicators (https://databank.worldbank.org/source/world-development indicators) and the International Monetary Fund (https://www.imf.org/en/Data). The researchers accessed the data by registering on the provided links. Throughout the study, the researchers ensured the confidentiality and privacy of the data.

## Empirical results and analysis

### Panel co integration and pooled mean group (PMG) ARDL analysis

According to [75], the panel data analysis provides a more precise point estimates of co-integrating vectors with reasonable accurate asymptotic approximations to the exact sampling distribution. Besides, the panel data provide more degrees of freedom added to a more conclusive and efficient estimates for highly indebted East African counters. In this study, the panel with integration results from Table 2 suggested that there is a long-run association among variables in the foreign direct investment (FDI) equation under all alternatives (Pedroni and Kao).

**Table 2:** panel co-integration results.

Pedroni	Statistic	Prob.
**Between dimension**		
Modified Phillips-Perron	0.0027	0.4989
Phillips-Perron	4.4726	0.0000
Augmented Dickey-Fuller	-5.3564	0.0000
**Within dimension**		
Modified variance ratio	-0.7588	0.2240
Modified Phillips-Perron	-0.8539	0.1966
Phillips-Perron	4.5061	0.0000
Augmented Dickey-Fuller	-5.0182	0.0000
**Kao**		
Modified Dickey-Fuller		0.0000
Dickey-Fuller	-11.0205	0.0000
Unadjusted modified Dickey-Fuller	11.2962	0.0000
Unadjusted Dickey-Fuller	-4.0393	0.0000

**Note: SIC** criteria used for lag selection

**Source:** Author’s estimation using **STATA 15**

After conducting a rigorous panel analysis, the study selected PMG approach. According to [73], the panel pooled mean group PMG/ARDL approach is used to estimate the long run and dynamic short run association between variables. The PMG estimates are presented in Table 3 and its result asserts the existence of long run association among variables, since the error correction term (adjustment coefficient) is negative and statically significant asserts. The panel estimate shows that market share and exchange rates have a positive short and long run statistical association with foreign direct investment inflow in a highly indebted eastern African nation.

**Table 3:** Panel long-run and dynamic short-run coefficients using PMG/ARDL model.

Variable	Coefficient	SE	Z	Probability
**Long run equation**				
LogMS	1.973102	.8263454	2.39	0.017
LogLF	-4.068063	2.820126	-1.44	0.149
LogED	-.089716	.25326	-0.35	0.723
LogNR	-.141327	.3098351	-0.46	0.648
LogINF	.0638166	.2859158	0.22	0.823
LogER	1.522705	.7642856	1.99	0.046
**Short run equation**				
**ECT(-1)**	**-.4421876**	.2120509	**-2.09**	0.037
Δ*LogMS*_−1_	.2527144	2.749311	0.09	0.927
Δ*LogLF*_−1_	10.43873	16.16605	0.65	0.518
Δ*LogED*_−1_	2.005598	1.978691	1.01	0.311
Δ*LogNR*_−1_	.2415789	.1840825	1.31	0.189
Δ*LogINF*_−1_	-.2725253	.2023387	-1.35	0.178
Δ*LogER*_−1_	2.518699	.4204556	5.99	0.000
Constant	7.518468	3.108078	2.42	0.016
Log Likelihood	-83.023			

Notes: Dependent variable: **DLogFDI:** Method: PMG = ARDL. **Sample:** 1990–2022. Maximum dependent lag: four (automatic selection). Model selection method: Schwarz criterion **(SIC).** Dynamic regressors (four lags, automatic): Selected model: **ARDL [1 1 1 1 1 1 1]**

**Source**: Author’s estimation using **STATA 15**

#### Market size and foreign direct investment

The result indicates the market size can attract FDI in selected eastern Africa nations. Potential markets can improve the elastic investor’s opportunity to maximize their economic scale. The study was consistent with [10, 37, 45, 46, 48, 50, 51, 53, 63–65, 76]. Furthermore, the study supports market imperfection theory, arguing that as a result of market imperfection, multinational corporations might base their businesses or manufacturing operations in these nations to capitalize on economies of scale [29] and the market size hypothesis [42] stressed that developed western foreign investors usually target an economy with large market. Likewise, the study supports Dunning (1995) Market-seeking (horizontal) entails replicating production facilities in the host country. These are tariff-jumping or export-substituting FDI utilized to better service a local market, although market size and expansion are crucial criteria for FDI. Furthermore, this result supports the market size hypothesis, introduced by [77] and later improved by [78] which postulated that a large market is the key for efficient use of resources and probe of economic scale.

Overall, this study suggests that market size lodges a precise vital place amid the determinate of FDI in flow and on the foundation, achieving a boost influence over investment decisions of MNCs in highly indebted eastern African nations.

### Exchange rate and foreign direct investment

Exchange rate devaluations has a positive statically long run association with FDI. The result of the study is consistent with [59, 79, 80]. Exchange rate key indicators of country intention competence in international markets. But currency devaluation can attract of FDI inflow if the purpose of attracting is to build an export application plant.

Currency devaluation is found to encourage inward foreign direct investment (FDI) as it makes the local currency cheaper. Multinational corporations (MNCs) prefer investing in markets where their currency can buy more. When deciding on FDI location and timing, investors take into account not only the current value of a local currency but also its expected fluctuations in the near future [76]. On the contrary to this study [81], suggested that the strongest yen in dollars and other Asian currencies promote Japan FDI and appreciation in the value of dollars arouse U.S firm FDI in Europe [82]. Since the host nation’s strong currency can maximize the wealth of MNCSS and minimize production cost [83] and reduce the costs of domestic assets and factors of production. Appreciation stimulates FDI, mainly when it stems from a widespread surge in capital flow or helps reduce protectionist pressures [84].

According to a study by [85], the impact of exchange rate fluctuations on foreign direct investment (FDI) remains uncertain. The real exchange rate is considered a measure of a nation’s global competitiveness. While a decrease in the real exchange rate is believed to attract more FDI, some argue that overpriced currency rates may not be a significant barrier unless FDI aims to serve as an export platform. Conversely, a decline in the exchange rate raises the prices of imported goods and reduces the value of profit transfers in foreign currency, negatively affecting the profitability of FDI projects. This effect is more pronounced when FDI primarily targets the domestic market. Therefore, if potential investors perceive changes in the exchange rate as indicative of future trends, it may influence their investment decisions.

When a production export-oriented multinational corporation experiences an increase in depreciation of the host nation’s currency, it positively impacts national input, production, exports, and profit due to the income effect. However, if the MNC heavily relies on imported materials for production, this depreciation can lead to reduced exports and profits, known as the cost effect. In such cases, if the domestic currency depreciates in foreign exchange markets, it negatively affects foreign direct investment (FDI). The influence of foreign exchange rates on FDI depends on the balance between the income effect and the cost effect. If the income effect is stronger, an increase in the exchange rate has a positive influence on FDI. Conversely, if the cost effect outweighs the income effect, a higher exchange rate has a negative impact on FDI [86, 87].

When a currency depreciates, its value decreases compared to another currency, and this has two potential effects on foreign direct investment (FDI). It lowers wages and production costs in the country, making it more attractive for foreign investors. This "relative wage" effect improves the overall return on investment for foreigners considering projects in that country. A depreciation of the currency in the target market increases the relative wealth of investors from the source country, resulting in higher acquisitions of assets. If investors from the source country hold more of their wealth in their own currency, a depreciation of the target currency enhances their relative wealth position and reduces their cost of capital. This allows them to make more competitive bids for assets abroad [58].

### Bound testing and ARDL analysis

The bound test results for the FDI models are presented in Table 4 and the result indicates that all variables have long-term associations in Kenya, Tanzania, and Rwanda. While the long-term association for Ethiopia is conclusive based bound testing the critical value. Furthermore, the result of ARD long run and dynamic short run estimates presented in Table 5 generally asserts a more or less similar results with panel pooled mean group (PMG) model estimation for all variables.

**Table 4:** Bound test results.

Country	F-test	T-test
Ethiopia	3.735	-3.914
Rwanda	4.507	-4.720
Tanzania	53.089	-8.370
Kenya	11.100	-6.789

**Notes:** Pesaran et al. (1998) bound testing critical values for F-test with k **(= 4)** are [3.15, 4.43], [3.13, 4.40], [3.14, 4.42] and [3.12, 4.45] 1% significance level, bound test critical values for t-test k (= 4) are [-2.86,-4.38] 5% significant level and [-3.43,-4.99], [-3.43,-4.99] and (-3.43–4.99) 1% significance level for others.

**Source:** Author’s estimation using **STATA 15**

**Table 5:** Short-run dynamics and long-run estimates using the error correction form of ARDL.

Country		Ethiopia	Kenya	Rwanda	Tanzania
**Long-run**					
LogMS		-.1325615	1.245296	2.162293***	2.983343***
logLF		9.688319	-3.735301	5.593051*	-8.704023***
LogED		.0468265	1.501835**	.1010221	.0098369
LogNR		-.9586908	-2.274488***	.7749853*	.1255487
LogINF		-.4273045	.2086938	-.1654137	.2536437
LogER		-2.082732	2.196582**	-1.053412***	.1209612
Constant		-149.1396*	5.528063	-47.71052***	31.15216 *
***ECT***_***t*−1**_		**-1.052432** ***	**-1.518643** ***	**-1.020569** ***	**-.7644165** ***
**Short-run**					
Δ*LogFDI*_−1_					
LD			.276567 *		
Δ*LogMS*_−1_		-2.639095	-1.280158	4.758456***	-2.581403**
LD		.0148412	3.540354**	3.675688***	-.1100985
Δ*LogLF*_−1_		18.23961	-12.9732		7.216296*
Δ*LogNR*_−1_		.5350452	2.469784 **		
LD			1.891472 ***		
Δ*LogINF*_−1_		-1.618048			
Δ*LogER*_−1_		-1.618048		3.401345	-4.909021***
Δ*LogED*_−1_			4.583761 **		
Constant		-149.1396*	5.528063	47.71052***	31.15216*
Residual diagnostics and model stability					
R^2^, adjusted R^2^	0.8547, 0.5640		0.904, 0.8217	0.9486, 0.9228	0.9676,0.9488
F stat. (Prob.)	0.0006		0.0000	0.0000	0.0000
Durbain Watson	2.647575		1.856259	1.989071	2.577721
White s test	0.4058		0.4154	0.4253	0.4514
Ramsey RESET test	0.3363		0.5282	0.1197	0.421
CUSUM	Pass		Pass	Pass	Pass
CUSUM^2^	Pass		Pass	Pass	Pass

Notes

*, **, ***Significant at 10, 5, and 1% significance levels, respectively

**Source:** Author’s estimation using **STATA 15**

The long-run short-run dynamic ARDL approach Table 5 indicates the exchange rate and external debt have a long run positive association with FDI in Kenya. On the other hand, the availability of natural resources is a negative impact on FDI in Kenya. Market size, labour force, and availability of natural resources have a positive statically (1%) long run relation with foreign direct investment in Rwanda, further market size and labour force have positive and negative association with FDI in Tanzania respectively.

#### Labour force and foreign direct investment

The short run dynamic and long run estimate of labor force as indicated in Table 5 has a positive and significant impact in the long run with FDI in Rwanda, while it may have a positive and negative association with FDI in the short run and long run respectively in Tanzania. The long-run association in Rwanda is supported by the location advantage of FDI, which states that low labor costs plays a prominent role in attracting FDI. The study’s findings are in line with those of [25, 44, 46, 65],who all came to the same conclusion that cheap labor does not significantly contribute to attracting FDI because foreign investors are primarily focused on labor productivity [44]. The study also supports resource-seeking (vertical) or export-oriented FDI, which invests in foreign nations to acquire resources such as raw materials, labor, and natural resources. Low-cost labor and abundant natural resources encourage FDI to achieve economies of scale and breadth [40].

While, the negative long-run association between labour force and FDI in Tanzania indicates that the labour force is not enough to attract MNCs. Rather, the skilled labour force with quality production makes forging investor business profitable. More recent data shows FDI in developing countries increasingly flows to medium and high-skilled manufacturing sectors, involving elevated income levels rather than cheap unskilled labor force, Furthermore [88], stated that Sri Lanka, India, and Vietnam are prominent examples for quality labor force, communication skills with large populations, earn high salaries, and they also become the means of existence of money garment industry.

Many countries in the early stages of development (from underdeveloped countries to developing countries) mainly use the competitive advantage of young and cheap labor, abundant and diversified natural resources, and many policy incentives to attract FDI flows. However, these advantages only last in the long run, and an increase in the number of laborers without an increase in labor quality will lead to the risk of a decline in FDI inflow. However, those nations have discovered that having unskilled labour no longer a competitive advantage in luring FDI to flow concurrently, firms need FDI to change technology lines in production, business, and co-operate governance. As a result, a quick transition from unskilled labour to skilled labour by altering the growth model and enhancing the caliber of human resource training aids in luring FDI [89].

Generally speaking, the availability of low-cost unskilled labor is a significant location-specific determinant of FDI in developing nations. The shift in foreign direct investment (FDI) from labor-intensive, low-cost, low-skill manufacturing to capital-, knowledge-, and skill-intensive industries is a result of new technological developments that have reduced the labor content of production and increased the knowledge content, making the availability of a pool of educated workers more desirable for multinational corporations than low labor costs alone [90].

#### External debt and FDI

Contrary to economic theory, which states that high external debt service increases tax and deters foreign investment, the study’s findings show that external debt may have a positive significant impact on Kenya’s FDI inflow both in the long and short term. As such, governments should make an effort to borrow from external sources because it has been found that external debt positively influences financial development [91]. Furthermore, the study, which was supported by proponents of the dual-gap theory, suggested the need for external source of finance like borrowing / donation. External debt has been widely advocated as an essential strategy used by most developing economies to attract funds to boost their economies. Due to its inability to generate sufficient funds domestically, a country like Kenya relies on external funds to enhance its economic growth and attract MNCs. This study postulates that The Kenyan economy has continued to rely on outside funding, which has resulted in the country having an excessive amount of debt; however, the country’s reliance on outside funding is promoted through its FDI inflows, which improve growth. The effective use of external funds results in infrastructural development, which in turn entices foreign investors. This is how foreign debt typically has a positive impact on foreign investment: when the government uses external funds appropriately, funds are used to develop the country’s infrastructure or to address its urgent economic needs.

### Availability of natural resources and FDI

Availability of natural resources may have a positive long-run association in Rwanda, the result of the study is consistent with consistent with [25, 36, 41, 70, 92] and proves Dunning (1995) resource-seeking FDI, where firms invest in foreign countries to access resources such as raw materials and natural resources. While positive (short run) and negative (long run) association with FDI in Kenya. The result is consistent with [3, 21, 67, 69], and Overall, the study MNCs is not only driven by natural resources but in the country but by some exogenous factors. As such, examining other political, institutional factors and sectoral analysis of FDI to be examined to reveal the mechanism of this impact.

## Conclusion

This study focuses on analyzing outward-looking FDI and its determinants in highly indebted developed countries in Eastern Africa. To achieve this objective, the study employed the pooled mean group (PMG) approach for panel data analysis across four countries and utilized bound testing and ARDL models for analyzing time-series data from the study period spanning 1990 to 2022 for each individual country in the sample.

The pooled mean group (PMG) approach indicated that both market share and exchange rates exert a significant impact on foreign direct investment (FDI) in both the short term and long term. Market size holds a significant position among the factors influencing the flow of (FDI) in developed eastern African countries. It exerts a strong influence on the investment decisions of (MNCs). The findings of the study align with the market imperfection theory and market size hypothesis, indicating that developed Western foreign investors typically prioritize economies with a large market. Additionally, the study highlights that East Africa is predominantly dominated by market-seeking (horizontal) MNCs, which have arrived to better serve the local market. Consequently, market size and expansion emerge as crucial criteria for this type of FDI.

Currency devaluation in developed nations is found to incentivize inward foreign direct investment (FDI) as investors tend to prefer investing in markets where their currency holds more purchasing power. When making decisions regarding FDI location and timing, investors consider not only the present value of the local currency but also its anticipated fluctuations in the near future. For production and export-oriented foreign investors, an increase in depreciation of the host nation’s currency has a positive impact on national input, production, exports, and profit due to the income effect. Host nation currency depreciation has two potential effects on FDI. Firstly, it reduces wages and production costs within the country, making it more appealing for foreign investors. This "relative wage" effect enhances the overall return on investment for foreign entities considering projects in that particular country.

The ARDL approach indicates that labour costs may have a positive long-term impact in Rwanda and suggests that Resource-seeking (vertical) or export-oriented FDI, which aims to acquire resources such as raw materials, labor, and natural resources in foreign countries, plays a significant role. The availability of low-cost labor and abundant natural resources encourages FDI and contributes to achieving economic scale in Rwanda.

Contrarily, in Tanzania, a cheap labor force is not enough to attract (MNCs) in the long run. A skilled labor force capable of high-quality production is essential for foreign investors to find business ventures profitable. Recent trends show that FDI in developing countries has shifted towards medium and high-skilled manufacturing sectors that provide higher income levels, rather than relying solely on cheap unskilled labor. Having an unskilled labor force no longer serves as a competitive advantage for attracting FDI. Hence, firms require FDI to upgrade technology, improve production lines, and enhance corporate governance.

The availability of natural resources has a positive impact in the short run but a negative impact in the long run in Kenya. The study suggests that other institutional variables need to be examined to provide a more conclusive analysis. On the other hand, external debt exhibits both short-term and long-term associations with FDI inflows in Kenya. External debt has been widely utilized by developing economies as a strategy to attract funds and stimulate economic growth. Kenya, relying heavily on external funding due to limited domestic resources, has accumulated a significant amount of debt. However, this reliance on outside funding has positively influenced FDI inflows and facilitated economic growth. The effective utilization of external funds has led to the development of robust infrastructure, which, in turn, attracts foreign investors.

### Recommendation policy maker and future researchers

Policymakers should prioritize market expansion and create a larger consumer base through initiatives like market development programs, trade agreements, and incentives for domestic and foreign companies to expand their market reach. They can leverage currency devaluation to attract more FDI, monitoring and managing currency fluctuations effectively. Policymakers in least developed Eastern African nations should undertake regular assessments of currency valuations and collaborate with monetary authorities to effectively manage exchange rate fluctuations. Moreover, fostering coordination among various government departments and agencies engaged in attracting and regulating Foreign Direct Investment (FDI) is essential. This cohesive approach ensures that policies are aligned and complementary. Additionally, it is crucial to establish clear communication channels between the government and the business community to address concerns and cultivate a conducive investment climate. Develop the capacity for skillful negotiation of investment treaties to ensure a fair balance between the interests of the host country and the expectations of foreign investors. Well-negotiated agreements can provide stability, protection, and incentives for FDI while safeguarding the nation’s sovereignty and economic interests. For future researchers, investigating the role of political and institutional variables and sectoral determinants of FDI across a larger panel of Sub-Saharan African countries in comparison with other regions is recommended.

## Abbreviations

FDI: Foreign Direct Investment
MNCs: Multinational companies
PMG: Pooled Mean Group
LDCs: Least Developed Countries
